# Effect of self-regulatory behaviour change techniques and predictors of physical activity maintenance in cancer survivors: a 12-month follow-up of the Phys-Can RCT

**DOI:** 10.1186/s12885-021-08996-x

**Published:** 2021-11-25

**Authors:** Anne-Sophie Mazzoni, Hannah L. Brooke, Sveinung Berntsen, Karin Nordin, Ingrid Demmelmaier

**Affiliations:** 1 Department of Public Health and Caring Sciences, Uppsala University, Uppsala, Sweden; 2 Department of Sport Science and Physical Education, University of Agder, Kristiansand, Norway

**Keywords:** Behaviour change, Behavioural support, Cancer survivors, Determinants, Exercise, Maintenance, Oncology, Self-regulation

## Abstract

**Background:**

Current knowledge about the promotion of long-term physical activity (PA) maintenance in cancer survivors is limited. The aims of this study were to 1) determine the effect of self-regulatory BCTs on long-term PA maintenance, and 2) identify predictors of long-term PA maintenance in cancer survivors 12 months after participating in a six-month exercise intervention during cancer treatment.

**Methods:**

In a multicentre study with a 2 × 2 factorial design, the Phys-Can RCT, 577 participants with curable breast, colorectal or prostate cancer and starting their cancer treatment, were randomized to high intensity exercise *with* or *without* self-regulatory behaviour change techniques (BCTs; e.g. goal-setting and self-monitoring) or low-to-moderate intensity exercise *with* or *without* self-regulatory BCTs. Participants’ level of PA was assessed at the end of the exercise intervention and 12 months later (i.e. 12-month follow-up), using a PA monitor and a PA diary. Participants were categorized as either maintainers (change in minutes/week of aerobic PA ≥ 0 and/or change in number of sessions/week of resistance training ≥0) or non-maintainers. Data on potential predictors were collected at baseline and at the end of the exercise intervention. Multiple logistic regression analyses were performed to answer both research questions.

**Results:**

A total of 301 participants (52%) completed the data assessments. A main effect of BCTs on PA maintenance was found (OR = 1.80, 95%CI [1.05–3.08]) at 12-month follow-up. Participants reporting higher health-related quality-of-life (HRQoL) (OR = 1.03, 95%CI [1.00–1.06] and higher exercise motivation (OR = 1.02, 95%CI [1.00–1.04]) at baseline were more likely to maintain PA levels at 12-month follow-up. Participants with higher exercise expectations (OR = 0.88, 95%CI [0.78–0.99]) and a history of tobacco use at baseline (OR = 0.43, 95%CI [0.21–0.86]) were less likely to maintain PA levels at 12-month follow-up. Finally, participants with greater BMI increases over the course of the exercise intervention (OR = 0.63, 95%CI [0.44–0.90]) were less likely to maintain their PA levels at 12-month follow-up.

**Conclusions:**

Self-regulatory BCTs improved PA maintenance at 12-month follow-up and can be recommended to cancer survivors for long-term PA maintenance. Such support should be considered especially for patients with low HRQoL, low exercise motivation, high exercise expectations or with a history of tobacco use at the start of their cancer treatment, as well as for those gaining weight during their treatment. However, more experimental studies are needed to investigate the efficacy of individual or combinations of BCTs in broader clinical populations.

**Trial registration:**

NCT02473003 (10/10/2014).

**Supplementary Information:**

The online version contains supplementary material available at 10.1186/s12885-021-08996-x.

## Background

There is strong evidence that regular physical activity (PA) improves many cancer-related health outcomes such as cancer-related fatigue, depression, quality of life and physical function [1]. Furthermore, emerging observational data suggest that regular PA can reduce cancer recurrence [2, 3] and increase overall survival [4]. Although cancer survivors have much to gain from regular PA, the majority tend to reduce their level of PA after being diagnosed [5, 6]. A PA decline is usually seen during the years following a cancer diagnosis [5], which indicates that engaging in PA during and after a cancer treatment is challenging for many cancer survivors. Therefore, many interventions have been designed to promote PA in this population and short-term PA increases have been demonstrated [7, 8]. However, sustaining this behaviour change is crucial to achieve long-term health benefits. PA maintenance, defined as a sustained PA behaviour for at least six months after the end of an intervention [9, 10], is therefore an area of particular interest in exercise oncology but is still insufficiently studied [7, 8].

Some systematic reviews have suggested that cancer survivors may benefit from behavioural interventions promoting long-term PA [8, 11]. Such interventions typically involve strategies to facilitate PA, including the use of behaviour change techniques (BCTs), defined as specific components of an intervention designed to change a behaviour [12]. A taxonomy of 93 BCTs has been developed by Michie et al. [12] to provide a standardised method of classifying such intervention components. Using this taxonomy, the above-mentioned reviews [8, 11] have reported that most interventions, targeting long-term PA, use different combinations of BCTs. Graded tasks, instructions on how to perform a specific PA behaviour and social support are examples of commonly used BCTs in interventions involving cancer survivors, irrespective of their efficacy in promoting PA [8, 11]. On the other hand, self-regulatory BCTs such as action planning, goal-setting and self-monitoring are frequently used in interventions involving different adult populations and resulting in high PA maintenance [8, 13]. Such BCTs are likely to facilitate *self-regulation* (i.e. ability to control/regulate one’s behaviour), which is a central concept in several theoretical frameworks such as Social Cognitive Theory [14] and has been identified as an important skill for successful PA maintenance [13]. Consequently, self-regulatory BCTs may be useful to maintain PA but more research is needed to confirm their possible impact on long-term PA maintenance in cancer survivors. We recently reported the effect of self-regulatory BCTs (e.g. goal-setting and self-monitoring) on cancer-related fatigue [15] and exercise adherence [16] in patients undergoing curative cancer treatment and participating in a six-month exercise intervention, the Physical Training and Cancer randomised controlled trial (Phys-Can RCT). We found no effect of self-regulatory BCTs on these outcomes during the intervention [15, 16]. However, determinants of PA behaviour in the short term are not necessarily the same in the long term [13]. Therefore, further investigations regarding the effect of self-regulatory BCTs on long-term PA are needed.

Additionally, increased knowledge about who may benefit most from behaviour change support is needed to develop more effective exercise interventions and optimize the use of such support in cancer survivors. For this purpose, it has been recommended to use a wide biopsychosocial approach [17]. This approach recognises the influence of multiple factors in PA behaviour change, including biological, psychological and social factors, and emphasizes that those different factors need to be considered to fully understand a PA behaviour [17]. Therefore, identifying predictors of long-term PA maintenance, based on physical, psychological and sociodemographic characteristics, both at baseline and post-intervention, is crucial to target vulnerable patient groups early in the course of their cancer treatment [18, 19] and after participating in an exercise intervention [20, 21]. Several factors such as body mass index (BMI) [22] and cardiovascular fitness [21, 22] (physical factors), exercise motivation [21, 22], exercise expectancy [22, 23] and exercise self- efficacy [10, 24] (psychological factors) as well as age [10, 22] and education [18] (sociodemographic factors) have been identified as potential predictors of PA maintenance. However, study findings are mixed and thus inconclusive. Furthermore, the majority of studies have examined predictors of short-term maintenance (e.g. 6 months post-intervention) [25], limiting the possibility to draw conclusions about predictors of PA maintenance in the longer term (12 months or more).

To date, knowledge about long-term PA maintenance after an exercise intervention in cancer survivors is limited. The aims of this study were therefore to 1) determine the effect of self-regulatory BCTs on long-term PA maintenance, and 2) identify baseline and post-intervention predictors of long-term PA maintenance in cancer survivors 12 months after participating in an exercise intervention during cancer treatment.

## Methods

### Study design and participants

This study is a secondary analysis using data from the Phys-Can RCT. The Phys-Can RCT is a multicentre study with a 2 × 2 factorial design (NCT02473003), approved by the Regional Ethical Review Board in Uppsala (Dnr 2014/249) [15, 26]. The Phys-Can RCT was designed to compare the effects of six months of high (HI) versus low-to-moderate intensity (LMI) exercise *with* or *without* self-regulatory BCTs on cancer-related fatigue in patients undergoing curative cancer treatment. All participants provided written informed consent. The recruitment process has previously been described in detail [15]. Briefly, eligible patients newly diagnosed with breast, colorectal or prostate cancer and scheduled to undergo (neo-)adjuvant cancer treatment, were recruited at university hospitals in three cities in Sweden (Lund/Malmö, Linköping and Uppsala) between March 2015 and April 2018. Participants were randomised to one of four intervention groups: 1) HI *with* self- regulatory BCTs, 2) LMI *with* self-regulatory BCTs, 3) HI *without* self-regulatory BCTs or 4) LMI *without* self-regulatory BCTs.

Participants included in the present study were those with PA data available at the end of the exercise intervention (6 months after randomization) and at 12-month follow-up (12 months after the end of the exercise intervention). Further, participants were included if they met international exercise guidelines for cancer survivors [1, 27] at the end of exercise the intervention (i.e. at least 75 min/week of vigorous intensity aerobic PA or 150 min/week of moderate intensity aerobic PA or 90 min/week of moderate-to-vigorous intensity aerobic PA, and/or two sessions of resistance training/week).

### Intervention

#### Exercise programme

The exercise programme has been described previously in detail [15]. Briefly, participants exercised for six months while undergoing cancer treatment. The exercise programme consisted of supervised group-based resistance training and individual home-based endurance training. The resistance training included six machine-based exercises and was performed twice a week. Participants alternated between 3 × 6 repetitions maximum (RM) and 3 × 10 RM in the HI groups, and 3 × 12 repetitions at 50% of 6 RM and 3 × 20 repetitions at 50% of 10 RM in the LMI groups. The endurance training consisted of twice-weekly interval training (20–40 min/session) at 80–90% of heart rate reserve (HRR) in the HI groups, and 150 min weekly continuous-based exercise at 40–50% of HRR in the LMI groups.

#### Self-regulatory BCTs

Participants randomised to receive self-regulatory BCTs were guided by study coaches to use goal-setting, review of behavioural goal, self-monitoring, action planning and problem solving as described in Table 1. These BCTs were provided face-to-face, jointly with the resistance training sessions on a maximum of nine occasions, except for self-monitoring which was performed by the participants after each exercise session. Weekly behavioural meetings were offered during the first month to provide participants with a gradual introduction to the use of the different BCTs. For example, the study coaches assisted participants in formulating concrete and realistic goals as well as exercise plans specifying when, where and how to perform endurance training that was home-based. After the first month, those meetings were held every 4–6 weeks, depending on individual needs. The study coaches used printed sheets during each meeting to make notes about what was decided (goal-setting, exercise planning). Those notes were then discussed during the next meeting, and if participants did not manage to exercise according to their planning, strategies to overcome barriers to exercise were identified and adjustments were made. Participants also developed an individual written plan for relapse prevention at the end of the exercise intervention, including goal-setting and coping planning. This plan was individually followed up and revised at three and nine months post-exercise intervention in face-to-face or telephone meetings with the study coaches (Table 1).

**Table 1:** Self-regulatory BCTs provided in the Phys-Can RCT

BCTs	Description	During the exercise intervention	After the exercise intervention
Goal-setting	Participants wrote specific weekly behavioural goals (exercise frequency, intensity, time and/or type) in electronic or printed training logs.	x	
Review of behavioural goals	Participants reviewed their behavioural goals with their study coaches to check if those goals were reached. Adjustments were made when needed.	x	
Self-monitoring	Participants recorded each training session in electronic or printed training logs. The logs included reflective notes regarding their exercise experiences, thoughts and feelings.	x	
Action planning	Participants made exercise plans with their study coaches specifying when, where and how to exercise.	x	
Problem solving	Participants analysed their training logs with the study coaches and identified strategies to overcome barriers. They also developed a plan for relapse prevention, including goal-setting and coping planning. This plan was written; one copy for the participant and one for the coach to follow-up.	x	
Follow-up prompts	Participants were followed up by their study coaches at 3 and 9 months after the exercise intervention. The individual plan for relapse prevention, including goal-setting and coping planning, was reviewed and revised at these occasions.		x

*BCTs* Behaviour change techniques, *Phys-Can* Physical Training and Cancer, *RCT* Randomised controlled trial

### Assessment of physical activity

PA was assessed at the end of the exercise intervention (6 months after randomisation) and at 12-month follow-up (12 months after the end of the exercise intervention), using previously validated instruments: a tri-axial PA monitor, the SenseWear Armband mini (SWA) [28, 29] and a 7-day PA diary [30]. At each measurement point, participants were asked to wear the SWA 24 h a day for seven consecutive days and fill out the diary during the same period.

To assess the weekly aerobic PA duration and intensity, data from the SWA were analysed with the SenseWear Professional 8.1 Software. The software provided SWA wear time and minutes spent in different levels of Metabolic Equivalent Task values (METs). PA at moderate and vigorous intensity was determined using established cut-off values, i.e. 3.0–5.9 METs and ≥ 6.0 METs respectively [31]. To reflect one week of PA, data from the SWA were included in the analyses if the SWA was worn for at least four days, including one weekend day, with a wear time of at least 80% per day [32–35]. Weekly time spent in the different intensity levels was calculated by summing minutes in 24 h for each valid day that the criterion for the relevant intensity was met.

To assess the weekly frequency of resistance training, data from the 7-day PA diary were used [30]. Participants made daily notes regarding the PA they performed, including type of activities. The weekly frequency of resistance training was determined by summing the number of times participants wrote in their diary activities such as “resistance training”, “resistance exercises” or “exercises on gym-machines”.

### Calculation of PA maintenance

PA maintenance was calculated as *level at 12-month follow-up minus level at the end of the exercise intervention.* This calculation was performed separately for moderate intensity aerobic PA, vigorous intensity aerobic PA and moderate-to-vigorous intensity aerobic PA (expressed in minutes/week) as well as for resistance training (expressed in number of sessions/week). The PA maintenance variable was dichotomised and participants were categorised as maintainers (i.e. change in any aerobic PA intensity level ≥ 0 and/or change in resistance training ≥0) or non-maintainers (i.e. maintained neither aerobic PA levels nor resistance training).

### Assessment of predictors

The choice and number of variables potentially associated with PA maintenance was based on previous research [18, 21, 25], and restricted by the sample size (i.e. at least five participants per predictor variable in the smallest group) [36]. A total of 12 variables were collected at baseline (prior to randomisation) and 11 at the end of the exercise intervention (6 months after randomisation). Variables were assessed with established and validated methods, and one study-specific item (see below).

#### Variables collected at baseline

Demographic and health behaviour information was collected by self-report and consisted of age, living situation (Living with a partner vs. Living alone) and tobacco use (Never smoked or used snus vs. Former or current smoker/snus user). Patient-reported variables were collected by self-reports and included anxiety assessed by the subscale of Hospital Anxiety and Depression Scale (HADS-A, range 0–21) [37], and cancer-related fatigue assessed by the subscale general fatigue of Multidimensional Fatigue Inventory (MFI-20, range 4–20) [38]. In the present study, the reliability of these two subscales was confirmed by Cronbach’s alpha values ranging from 0.86 to 0.87. Health-related quality-of-life (HRQoL) was assessed by the subscale of European Organisation for Research and Treatment of Cancer quality of life questionnaire (EORTC QLQ-C30, range 0–100) [39], and exercise self-efficacy by the 9-item Exercise Barrier Self-Efficacy Scale (EBSS, range 0–10) [40]. In the present study, the reliability of these scales was confirmed by Cronbach’s alpha values ranging from 0.85 to 0.87 for the subscale of EORTC QLQ-C30 and 0.90 to 0.91 for exercise self-efficacy. Exercise expectations were assessed with one study-specific item, where participants rated on a 0–10 numerical rating scale how confident they were that exercise could reduce the risk of cancer recurrence (0=‘Not at all confident’ and 10 = ‘Very confident’). We adapted this item from a previously validated template (weighted Kappa coefficient of 0.62) for adults with rheumatoid arthritis [41, 42]. Exercise motivation was assessed with a study-specific questionnaire including three items [43], where participants rated on a 100 mm visual analogue scale 1) how important it was to them 2) how confident, and 3) how ready they were to perform aerobic PA at moderate intensity (0=‘Not at all important/confident /ready’ and 100 = ‘Very important/confident/ready”). The three items were averaged to calculate an overall score. In the present study, the reliability of the overall score was confirmed by Cronbach’s alpha values ranging from 0.83 to 0.90. Objectively assessed variables consisted of cardiorespiratory fitness measured as maximal oxygen uptake (VO_2_max, mL/kg/min) during walking/running on a treadmill to exhaustion using a modified Balke-protocol [44], and body mass index (BMI, kg/m^2^) calculated by the study staff the day the VO_2_max test was performed. Data about (neo-)adjuvant cancer treatment (Chemotherapy vs. No chemotherapy) were extracted from medical records.

#### Variables collected post-exercise intervention

Patient-reported variables consisted of anxiety, cancer-related fatigue, HRQoL, exercise self-efficacy and exercise motivation, and were assessed as described above. Objectively assessed variables consisted of VO_2_max, BMI and muscle strength. VO_2_max and BMI were assessed as described above. Upper and lower muscle strength (kg) were assessed as one repetition maximum including chest press and seated leg press, respectively. Change scores for VO_2_max, BMI and muscle strength were used for the analyses and were computed by subtracting the baseline score from the post-exercise intervention score. Exercise adherence to the intervention was assessed using training logs filled out by the participants after each session and was calculated for resistance and endurance training respectively, as performed exercise volume divided by prescribed exercise volume (proportion, %) [15].

### Statistical analyses

Baseline characteristics across intervention groups were compared using one-way ANOVA or Kruskal-Wallis test for continuous variables and Chi^2^ test for nominal variables. Difference in baseline characteristics between those included in the analysis and those who were lost to follow-up were examined using independent t-test or Mann-Whitney test for continuous variables and Chi^2^ test for nominal variables.

PA maintenance at 12-month follow-up is reported using frequencies and proportions. The main effect of self-regulatory BCTs on PA maintenance was examined using multiple logistic regression. The analysis was adjusted for exercise intensity, interaction (exercise intensity x self-regulatory BCTs), study site and cancer diagnosis. All the variables were included in the model using effect coding [45], except for study site and cancer diagnosis that were included using dummy coding. The results are presented as odds ratio (OR) with 95% Confidence Intervals (95% CI).

Predictors of PA maintenance were examined using multiple logistic regression. Two models were run separately; one including baseline variables (Model 1) and one including post-exercise intervention variables (Model 2). Each model also included the intervention groups to adjust for potential differences between the groups. For each model, the variables were entered simultaneous [46]. Dichotomous variables were generated for nominal variables with two categories (i.e. living situation, tobacco use and cancer treatment). Age, anxiety, cancer-related fatigue, HRQoL, exercise self-efficacy, exercise expectations, exercise motivation, VO_2_max, BMI, muscle strength and exercise adherence were treated as continuous variables with ORs reported per whole unit of the measure (e.g. 1 year, 1 kg/m^2^ etc). Missing data were ≤ 7% in all variables, except for tobacco use (9.6% missing), VO2max change (10.6% missing) and muscle strength change (12.6% missing). Missing data were not associated with the majority of the baseline characteristics (e.g. age, living situation, education, BMI) or the outcome (PA maintenance) (Suppl. Table 1). Thus, participants with missing data were excluded by listwise deletion from the analysis as this statistical technique is considered to produce unbiased estimates of the exposure-outcome association if missing data are unrelated to the outcome [47]. The results are presented for each model separately. ORs with 95% CI are presented for each variable and indicate the individual contribution of each variable after adjusting for all the others included in the same model.

Assumptions of linearity (continuous variables only), non-multicollinearity (all variables) and goodness of fit were assessed for each analysis. All data analyses were performed with the Statistical Package for the Social Sciences (SPSS, v.27).

## Results

### Participants

Among the 577 participants randomised to one of the four intervention groups, 316 (55%) provided available SWA and diary data at the two measurement points (i.e. end of the exercise intervention and 12-month follow-up). Of those participants, 301 (95%) performed the required minimum level of PA at the end of the exercise intervention and were included in the analyses.

Participants’ characteristics were similar in the four intervention groups (Table 2). At baseline, participants’ mean age was 59 years (SD 12). The majority were diagnosed with breast cancer (78%) and received chemotherapy as primary (neo-)adjuvant treatment (59%).

**Table 2:** Baseline characteristics of follow-up participants and participants lost to follow-up in the Phys-Can RCT

	Follow-up participants (***n*** = 301)				Participants lost to follow-up (***n*** = 276)	***P***-value ^**a**^
	Groups with BCTs		Groups without BCTs		All groups	
	HI (*n* = 77)	LMI (*n* = 81)	HI (*n* = 71)	LMI (*n* = 72)		
**Age**, mean (SD)	60 (12)	58 (12)	57 (11)	60 (11)	59 (12)	0.907
**Women**, *n* (%)	61 (79)	64 (79)	56 (79)	57 (79)	227 (82)	0.341
**Living situation,***n* (%)						
Living with partner	57 (75)	63 (82)	55 (78)	54 (75)	202 (79)	0.094
**Education**, *n* (%)						
University or equivalent	45 (59)	58 (73)	49 (69)	46 (64)	150 (57)	0.070
**Tobacco use,***n* (%)						
Former or current smoker/snus user	24 (35)	23 (32)	26 (40)	30 (44)	121 (50)	**0.006**
**Anxiety (0–21)**^**b,**^ mean (SD)	5 (4)	5(4)	6 (5)	5 (4)	6 (5)	0.451
**Cancer-related fatigue (4–20)**^**b**^, mean (SD)	11 (4)	11 (4)	11 (5)	11 (5)	12 (4)	0.248
**HRQoL (0–100)**^**b**^**,** mean (SD)	70 (20)	66 (19)	70 (20)	68 (19)	63 (21)	**0.005**
**Exercise self-efficacy (0–10)**^**c**^**,** mean (SD)	6 (2)	6 (2)	6 (2)	5 (1)	5 (2)	0.055
**Exercise expectations (0–10)**^**c**^, mean (SD)	6 (3)	6 (3)	6 (3)	6 (3)	6 (3)	0.674
**Exercise motivation (0–100)**^**c**^, mean (SD)	81 (23)	80 (25)	83 (19)	81 (19)	83 (19)	0.689
**Comorbidities**, *n* (%)						
One or more	37 (53)	35 (50)	37 (60)	38 (58)	146 (62)	0.087
**VO**_**2**_**max,** mean mL/kg/min (SD)	31 (8)	31 (7)	32 (6)	30 (7)	28 (7)	**< 0.001**
**BMI**, mean kg/m^2^ (SD)	26 (4)	25 (4)	25 (4)	25 (4)	27 (5)	**< 0.001**
**MVPA,** median min/week (IQR)	424 (260)	429 (386)	522 (417)	437 (353)	321 (331)	**< 0.001**
**Meeting exercise guidelines**^**d**^, n (%)	68 (99)	72 (97)	60 (98)	65 (97)	192 (91)	**0.001**
**Diagnosis**, *n* (%)						0.323
Breast cancer	60 (78)	63 (78)	56 (79)	56 (78)	222 (80)	
Prostate cancer	14 (18)	15 (18)	12 (17)	15 (21)	41 (15)	
Colorectal cancer	3 (4)	3 (4)	3 (4)	1 (1)	13 (5)	
**Primary (neo-)adjuvant treatment**, *n* (%)						0.284
Chemotherapy	42 (55)	43 (53)	37 (52)	35 (49)	141 (59)	
Radiation therapy	23 (30)	27 (33)	24 (34)	26 (36)	72 (30)	
Endocrine therapy	12 (16)	11 (14)	10 (14)	11 (15)	27 (11)	

^a^*p*-value for differences between participants included in the analysis and those who were lost to follow-up (independent t-test or Mann-Whitney for continuous variables and Chi2 test for nominal variables),^b^Higher scores indicate worse outcome, ^c^Higher scores indicate better outcome, ^d^guidelines for cancer survivors, i.e. at least 75 min/week of vigorous intensity aerobic physical activity or 150 min/week of moderate intensity aerobic physical activity or 90 min/week of moderate-to-vigorous intensity aerobic physical activity, and/or two sessions of resistance training/week. *Phys-Can* Physical Training and Cancer, *RCT* Randomised controlled trial, *BCTs* self-regulatory behaviour change techniques, *HI* High intensity exercise, *LMI* Low-to-moderate intensity exercise, *SD* Standard deviation, *HRQoL* Health-related quality-of-life, *VO*_*2*_*max* Maximal oxygen uptake, *BMI* Body mass index, *MVPA* Moderate-to-vigorous intensity physical activity, *IQR* Interquartile range. n’s do not all sum to total due to missing data; % is of those with available data

The 276 participants (48%) lost to follow-up significantly differed from the follow-up population at baseline in being mainly former or current smokers/snus users (*p* = 0.006), less physically active (*p* < 0.001), having a lower VO_2_max (*p* < 0.001), a lower HRQoL (*p* = 0.005), and a higher BMI (*p* < 0.001).

### PA maintenance at 12-month follow-up

The proportion of participants who maintained their PA levels at 12-month follow-up is provided for each intervention group in Table 3. In total, 223 participants (74%) maintained their level of PA at 12-month follow-up, either aerobic PA only (68%), resistance training only (2%) or both aerobic PA and resistance training (4%).

**Table 3:** Physical activity maintenance at 12-month follow-up in the Phys-Can RCT (*n* = 301)

	Groups with BCTs (***n*** = 158)		Groups without BCTs (***n*** = 143)	
	HI (*n* = 77)	LMI (*n* = 81)	HI (*n* = 71)	LMI (*n* = 72)
**Maintainers**, *n* (%) ^a^				
Aerobic only	61 (79)	54 (67)	40 (56)	51 (71)
Moderate intensity	35 (45)	30 (37)	28 (39)	27 (38)
Vigorous intensity	47 (61)	46 (57)	26 (37)	42 (58)
Moderate-to-vigorous intensity	35 (45)	29 (36)	26 (37)	27 (38)
Resistance only	2 (3)	1 (1)	2 (3)	0 (0)
Both aerobic and resistance	3 (4)	4 (5)	4 (6)	1 (1)
**Non-maintainers**, *n* (%)				
Neither aerobic nor resistance	11 (14)	22 (27)	25 (35)	20 (28)

^a^Participants who maintained their level of physical activity (aerobic and/or resistance) at 12-month follow-up in relation to post-intervention levels

*Phys-Can* Physical Training and Cancer, *RCT* Randomised controlled trial, *BCTs* Self-regulatory behaviour change techniques, *HI* High intensity exercise, *LMI* Low-to-moderate intensity exercise

### Effect of self-regulatory BCTs on PA maintenance

The multiple ordinal logistic regression revealed a main effect of self-regulatory BCTs on PA maintenance at 12-month follow-up (OR = 1.80, 95%CI [1.05–3.08], *p* = 0.033). A significant interaction was observed between exercise intensity and self-regulatory BCTs (OR = 1.77, 95%CI [1.03–3.04], *p* = 0.037), indicating that the effect of self-regulatory BCTs was larger in participants in the HI group *with* self-regulatory BCTs compared to those in the LMI group *with* self-regulatory BCTs.

### Baseline predictors of PA maintenance (Model 1)

Baseline predictors of PA maintenance are presented in Table 4. Sixty-three participants were excluded from the analysis due to missing data (Suppl. Table 1). Participants reporting higher HRQoL (OR = 1.03, 95%CI [1.00–1.06], *p* = 0.018) or higher exercise motivation (OR = 1.02, 95%CI [1.00–1.04], *p* = 0.011) at baseline were more likely to maintain their PA levels at 12-month follow-up. In contrast, participants reporting higher expectations to reduce the risk of cancer recurrence with exercise (OR = 0.88, 95%CI [0.78–0.99], *p* = 0.040) or being former or current smokers/snus users (OR = 0.43, 95%CI [0.21–0.86], *p* = 0.018) at baseline were less likely to maintain their PA levels at 12-month follow-up.

**Table 4:** Model 1- multiple ordinal logistic regression model estimating baseline predictors of physical activity maintenance^a^ (*n* = 238)

Baseline predictors		OR (95% CI)	***P-***value
Age	Per 1 year	1.00 (0.96–1.05)	0.846
Living situation	Living with partner	1.00	
	Living alone	0.63 (0.30–1.30)	0.213
Tobacco use	Never smoked or used snus	1.00	
	Former or current smoker/snus user	**0.43 (0.21–0.86)**	**0.018**
Anxiety	Per 1 unit on a 0–21 scale	1.09 (0.99–1.20)	0.098
Cancer-related fatigue	Per 1 unit on a 4–20 scale	1.04 (0.94–1.15)	0.455
HRQoL	Per 1 unit on a 0–100 scale	**1.03 (1.00–1.06)**	**0.018**
Exercise self-efficacy	Per 1 unit on a 0–10 scale	1.17 (0.97–1.42)	0.099
Exercise expectations	Per 1 unit on a 0–10 scale	**0.88 (0.78–0.99)**	**0.040**
Exercise motivation	Per 1 unit on a 0–100 scale	**1.02 (1.00–1.04)**	**0.011**
VO_2_max	Per 1 mL/kg/min	0.95 (0.89–1.01)	0.070
BMI	Per 1 kg/m^2^	1.02 (0.93–1.12)	0.706
Chemotherapy	No	1.00	
	Yes	0.83 (0.38–1.83)	0.651
Randomisation groups	HI *with* BCTs	1.00	
	HI *without* BCTs	**0.21 (0.07–0.56)**	**0.002**
	LMI *with* BCTs	0.46 (0.17–1.25)	0.126
	LMI *without* BCTs	0.43 (0.16–1.19)	0.104

^a^Categorized as maintainers or non-maintainers at 12-month follow-up

The results indicate the individual contribution of each variable after adjusting for all the other variables included in the model. *OR* Odds ratio, *CI* Confidence interval, *HRQoL* Health-related quality-of-life, *VO*_*2*_*max* Maximal oxygen uptake, *BMI* Body mass index, *HI* High intensity exercise, *BCTs* Self-regulatory behaviour change techniques, *LMI* Low-to-moderate intensity exercise. Bold values indicate *p*-values below 0.050

### Post-exercise intervention predictors of PA maintenance (Model 2)

Post-exercise intervention predictors of PA maintenance are presented in Table 5. Ninety-six participants were excluded from the analysis due to missing data (Suppl. Table 1). Participants who had greater BMI increases over the course of the exercise intervention (OR = 0.63, 95%CI [0.44–0.90], *p* = 0.010) were less likely to maintain their PA levels at 12-month follow-up.

**Table 5:** Model 2- multiple ordinal logistic regression model estimating post-exercise intervention predictors of physical activity maintenance^**a**^ (*n* = 205)

Post-exercise intervention predictors		OR (95% CI)	***P-***value
Anxiety	Per 1 unit on a 0–21 scale	1.06 (0.94–1.20)	0.309
Cancer-related fatigue	Per 1 unit on a 4–20 scale	0.99 (0.88–1.12)	0.877
HRQoL	Per 1 unit on a 0–100 scale	1.01 (0.97–1.03)	0.928
Exercise self-efficacy	Per 1 unit on a 0–10 scale	0.94 (0.77–1.14)	0.514
Exercise motivation	Per 1 unit on a 0–100 scale	1.00 (0.98–1.02)	0.923
VO_2_max change^b^	Per 1 mL/kg/min	0.99 (0.90–1.10)	0.938
BMI change^b^	Per 1 kg/m^2^	**0.63 (0.44–0.90)**	**0.011**
Upper muscle strength change^b^	Per kg	0.95 (0.88–1.04)	0.284
Lower muscle strength change^b^	Per kg	1.03 (0.98–1.08)	0.252
Adherence to the intervention (resistance training)	Per %	1.00 (0.98–1.03)	0.806
Adherence to the intervention (endurance training)	Per %	1.00 (0.99–1.02)	0.521
Randomisation groups	HI *with* BCTs	1.00	
	HI *without* BCTs	**0.35 (0.13–0.95)**	**0.040**
	LMI *with* BCTs	0.87 (0.30–2.56)	0.802
	LMI *without* BCTs	0.46 (0.17–1.25)	0.126

^a^Categorized as maintainers or non-maintainers at 12-month follow-up, ^b^ Change scores were computed by subtracting the baseline score from the post-exercise intervention score. The results indicate the individual contribution of each variable after adjusting for all the other variables included in the model. *OR* Odds ratio, *CI* confidence interval, *HRQoL*: Health-related quality-of-life, *VO*_*2*_*max* Maximal oxygen uptake, *BMI* Body mass index, *HI* High intensity exercise, *BCTs* Self-regulatory behaviour change techniques, *LMI* Low-to-moderate intensity exercise. Bold values indicate *p*-values below 0.050

## Discussion

To the best of our knowledge, this is the first study to examine both the effect of self-regulatory BCTs and predictors of long-term PA maintenance in cancer survivors after participating in an exercise intervention. We found that self-regulatory BCTs improved PA maintenance at 12-month follow-up in our study sample, especially in participants exercising at high intensity during the intervention. Further, higher HRQoL and higher exercise motivation at baseline were positively associated with PA maintenance at 12-month follow-up. In contrast, higher exercise expectations, being former or current smokers/snus users at baseline and gaining weight during the exercise intervention were negatively correlated with PA maintenance at 12-month follow-up.

Twelve months after the Phys-Can RCT, the odds of maintaining PA among participants receiving self-regulatory BCTs was 1.8 times the odds of maintaining PA among those who did not receive self-regulatory BCTs. This demonstrates the value of behavioural support to help cancer survivors in maintaining their PA levels in the long term after participating in an exercise intervention. Our results are in line with findings from two recent systematic reviews [8, 13], reporting that self-regulatory BCTs are effective in improving PA maintenance in cancer survivors [8] and in overweight and obese adults [13]. Our findings are also supported by a systematic review involving healthy and clinical adult populations [11], where the use of follow-up prompts (i.e. brief post-intervention contacts to reinforce previous intervention content) was a key strategy to achieve PA maintenance. Thus, it appears that patients are more likely to maintain their PA behaviour if their self-regulatory skills are developed or reinforced during the behaviour change process, which can be achieved using for example goal-setting, review of the behaviour and self-monitoring. Notably, the results from the present study differ from our previous findings, where no effect of the self-regulatory BCTs on exercise adherence was found during the exercise intervention [16]. This lack of effect could be explained by the intervention groups (*with* and *without* self-regulatory BCTs) being too similar during the exercise intervention as all were provided with support such as supervised training, social support and feedback. Thus, self-regulatory BCTs as additional support may not have been sufficient to make a difference during the exercise intervention. Another possible explanation is that the self-regulatory BCTs used in the Phys-Can RCT may be more effective in maintaining PA rather than adopting the behaviour [13]. Finally, the large number of lost to follow-up in the present study could also explain the different results, by introducing a selection bias (e.g. participants at follow-up could be those who were more motivated and receptive to this behavioural support). However, it is important to note that our study design does not allow us to determine which self-regulatory BCTs were the most effective in improving long-term PA maintenance in cancer survivors, highlighting the need for further research in broader clinical populations. Furthermore, the majority of our participants did not maintain their level of resistance training at follow-up. It reflects the challenges faced by cancer survivors to continue with this type of PA on their own following a supervised exercise programme, irrespective of the behavioural support provided. Similarly, results from previous studies show a decrease of PA levels following the completion of structured exercise interventions that offered supervised exercise [18, 21, 22, 24]. Besides treatment side-effects, barriers to exercise such as limited access to gym facilities, costs and lack of time are often reported in cancer survivors [48] and could explain the low maintenance levels post-intervention in our study. Therefore, providing participants at the end of exercise interventions with a more individually tailored prescription of resistance training, based on their preferences and needs, could be an adequate alternative to improve long-term PA maintenance of resistance training. For example, exercise barriers such as limited access to gym facilities, costs and lack of time could be overcome with a home-based resistance training programme. The choice of exercise type (e.g. focus on specific muscle groups or more functional exercises) and intensity (e.g. use of weights/resistance bands or merely body weight) should also be discussed with each participant based on their preferences and current capacities. The same principles should be applied regarding exercise frequency and duration with a stepwise progression of exercise.

Regarding predictors, our findings on HRQoL and exercise motivation are in line with the few previous studies investigating long-term PA maintenance in cancer populations. These studies found higher HRQoL [20] and higher exercise motivation [21] to predict PA maintenance in breast cancer survivors at 12-month, 24-month [21], and 5-year [20] follow-up. However, we found only weak associations between HRQoL /exercise motivation and maintenance compared to previous studies, and only regarding the variables at baseline. This could be explained by the high levels and small variation in HRQoL and exercise motivation in our study sample, limiting the possibility to examine the predictive value of these variables on PA maintenance. Notably, exercise self-efficacy did not predict long-term PA maintenance in our sample, which differs from previous studies [10, 24], and could also be explained by the small variation in exercise self-efficacy observed in our study sample. Another explanation is that most of the studies have examined the association between exercise self-efficacy and short-term PA maintenance (less than 12 months) [10, 24] in contrast to our study that focused on long-term PA maintenance. Indeed, findings from previous research indicate that predictors of PA behaviour may differ across the different phases of the behaviour change process (e.g. PA initiation, adoption, short/long-term maintenance) [49]. Interestingly, we found a negative association between baseline exercise expectations and long-term PA maintenance. It is possible that some participants may have had unrealistic expectations to be cured from the disease or feel healthy soon by exercising regularly but were still on treatment or still suffering from treatment-related symptoms at 12-month follow-up. This could have undermined their motivation to continue to be physically active at follow-up. This phenomenon is referred to as the *false hope syndrome* [50] and implies that high initial exercise expectations may undermine PA maintenance if the expected benefits are small or not attained [50, 51]. However, it is important to note that the item used in this study to measure exercise expectations has been adjusted from a previously validated template, although not validated in cancer survivors [41]. This could partly have impacted on our results and limit the interpretation of our findings. Consequently, given the inconsistent findings across the literature [52], further research is needed to investigate this association. Tobacco use was one of our strongest baseline predictors. This is in contrast with findings from previous studies that have reported no association [21] or borderline associations between smoking status and PA maintenance in cancer survivors [22, 53]. Those divergent results could be explained by the way this variable is usually categorised. In our study, current and former smokers were included in the same category, while in other studies, the variable is often dichotomised as current smokers/non-smokers, which limits the exploration of history of tobacco use as a predictor. In previous studies involving non-cancer populations [54–56], smokers and former smokers were found to have poorer health habits than non-smokers, which is partly consistent with our results. Change in BMI during the six-month exercise intervention was another strong predictor, indicating that participants who gained weight during their treatment were less likely to maintain the PA levels at follow-up. Weight gain is a common problem for many cancer survivors, especially for breast cancer survivors treated with adjuvant chemotherapy [57], and several studies have reported that higher weight/BMI is associated with lower exercise levels in cancer survivors [25]. It has been suggested that weight gain can render PA more challenging and less enjoyable to perform [53, 58]. It is also instructive to know that variables that were baseline predictors of PA maintenance in our study, were not post-exercise intervention predictors. This is, for example, the case regarding exercise motivation and could be explained by the measurement method (i.e. we asked our participants about motivation to perform exercise rather than motivation to maintain PA levels) and our definition of maintenance.

Our results may contribute to the development of more effective exercise interventions designed to promote long-term PA behaviour, and guide health professionals working within oncology healthcare. Indeed, this study provides valuable information, describing concrete tools (i.e. specific self-regulatory BCTs) to facilitate long-term PA maintenance in cancer survivors, and identifying patient groups who may benefit from such support. The strengths of the present study include the use of data from a large multicentre RCT, the long-term follow-up and the combination of objective and self-report PA data to capture different aspects of PA [30]. The present study also has limitations, including the large number of participants lost to follow-up and the homogeneous study sample (i.e. mainly women with breast cancer, highly educated, physically active and motivated to exercise), limiting the generalisability of our results. Further, the small number of participants who performed resistance training at 12-month follow-up did not allow us to analyse predictors of aerobic PA and resistance training separately. The outcome (PA maintenance) was therefore dichotomized (maintainers vs. non-maintainers), regardless of the type of PA maintenance (aerobic or resistance). This dichotomisation was deemed adequate as current guidelines stipulate that cancer survivors should perform either aerobic PA or resistance training to achieve similar health benefits [1]. Finally, only participants meeting the exercise guidelines at the end of the exercise intervention were included in the analyses. Using these PA cut-off values has limitations as participants who may have increased their PA levels after the exercise intervention were excluded. However, these criteria were applied to limit selection bias and exclude participants who could otherwise have been categorized as maintainers at 12-month follow-up despite low PA levels at the end of the exercise intervention. It is also important to keep in mind that 95% of participants with available PA data were included in the analyses, indicating a limited impact of applying such PA cut-off values on our results.

## Conclusion

Self-regulatory BCTs improved PA maintenance at 12-month follow-up in our study participants. The use of such behavioural support can therefore be recommended to cancer survivors for long-term PA maintenance. Patients with low HRQoL, low exercise motivation, high exercise expectations or with an history of tobacco use at the start of their cancer treatment, as well as those gaining weight during their treatment may be those most in need of such support to maintain PA. However, it remains unclear if similar results would be obtained in more heterogeneous populations and which self-regulatory BCTs are the most effective in improving this complex behaviour. More studies with experimental designs with long-term follow are therefore needed to investigate the efficacy of individual or combinations of BCTs in broader cancer populations.

## Supplementary Information

**Additional file 1: Suppl Table 1.** Physical activity maintenance at 12-month follow-up and baseline characteristics of participants included vs. excluded from the predictor analyses.

## Data Availability

The data that support the findings of this study are available upon reasonable request.

## Abbreviations

BCT: Behaviour change technique
BMI: Body mass index
CI: Confidence interval
EBSS: Exercise Barrier Self-Efficacy Scale
EORTC QLQ-C30: European Organisation for Research and Treatment of Cancer quality of life questionnaire C-30
HADS-A: Hospital Anxiety and Depression scale (Anxiety subscale)
HI: High intensity exercise
HRQoL: Health-related quality of life
HRR: Heart rate reserve
LMI: Low-to-moderate intensity exercise
MET: Metabolic Equivalent
MFI: Multidimensional Fatigue Inventory
OR: Odds ratio
PA: Physical activity
Phys-Can: Physical Training and Cancer
RCT: Randomised controlled trial
RM: Repetition maximum
SWA: SenseWear Armband mini
VO_2_max: Maximal oxygen uptake
