# Supplementary material for: Effect of self-regulatory behaviour change techniques and predictors of physical activity maintenance in cancer survivors: a 12-month follow-up of the Phys-Can RCT
Source: BMC Cancer. 2021 Nov 25;21:1272. doi: 10.1186/s12885-021-08996-x (PMC8613944; doi:10.1186/s12885-021-08996-x)
Supplement: Supplementary file 1 — Additional file 1: Suppl Table 1. Physical activity maintenance at 12-month follow-up and baseline characteristics of participants included vs. excluded from the predictor analyses. [file 12885_2021_8996_MOESM1_ESM.docx]

**Suppl. Table 1.** Physical activity maintenance at 12-month follow-up and baseline characteristics of participants included vs. excluded from the predictor analyses**.**

|  | **Model 1** | | **p-value ^a^** | **Model 2** | | **p-value ^a^** |  |
| --- | --- | --- | --- | --- | --- | --- | --- |
|  | **Participants included**  **(n =238)** | **Participants excluded**  **(n = 63)** |  | **Participants included**  **(n= 205)** | **Participants excluded**  **(n=96)** |  |  |
| **PA maintenance at 12-month follow-up**, *n* (%) |  |  |  |  |  |  |  |
| Maintainers | 173 (73) | 50 (78) | 0.520 | 150 (73) | 73 (76) | 0.673 |  |
| **Age**, mean (SD) | 58 (12) | 61 (12) | 0.173 | 58 (12) | 60 (11) | 0.130 |  |
| **Women**, *n* (%) | 196 (82) | 42 (67) | **0.005** | 165 (80) | 73 (76) | 0.447 |  |
| **Living situation,** *n* (%) |  |  |  |  |  |  |  |
| Living with partner | 182 (77) | 47 (80) | 0.730 | 162 (79) | 67 (73) | 0.231 |  |
| **Education**, *n* (%) |  |  |  |  |  |  |  |
| University or equivalent | 156 (66) | 42 (69) | 0.761 | 140 (68) | 58 (62) | 0.355 |  |
| **Tobacco use,** *n* (%) |  |  |  |  |  |  |  |
| Former or current smoker/snus user | 85 (36) | 18 (51) | 0.093 | 69 (36) | 34 (43) | 0.338 |  |
| **Anxiety (0-21)^b,^** mean (SD) | 5 (4) | 5 (4) | 0.746 | 5 (4) | 6 (4) | 0.282 |  |
| **Cancer-related fatigue (4-20)^b^**, mean (SD) | 11 (4) | 11 (5) | 0.778 | 11 (5) | 11 (4) | 0.851 |  |
| **HRQoL (0-100)^b^,** mean (SD) | 69 (19) | 66 (21) | 0.459 | 69 (19) | 67 (21) | 0.390 |  |
| **Exercise self-efficacy (0-10)^c^,** mean (SD) | 6 (2) | 6 (2) | 0.428 | 6 (2) | 6 (2) | 0.308 |  |
| **Exercise expectations (0-10)^c^**, mean (SD) | 6 (3) | 5 (3) | 0.215 | 6 (3) | 6 (3) | 0.892 |  |
| **Exercise motivation (0-100)^c^**, mean (SD) | 82 (21) | 79 (24) | 0.316 | 81 (22) | 83 (21) | 0.326 |  |
| **Comorbidities**, *n* (%) |  |  |  |  |  |  |  |
| One or more | 118 (55) | 29 (53) | 0.762 | 99 (53) | 48 (58) | 0.428 |  |
| **VO_2_max,** mean mL/kg/min (SD) | 31 (7) | 32 (7) | 0.379 | 32 (7) | 29 (7) | **0.012** |  |
| **BMI**, mean kg/m^2^ (SD) | 25 (3) | 25 (4) | 0.989 | 25 (4) | 26 (4) | 0.063 |  |
| **MVPA,** median min/week (IQR) | 440 (335) | 433 (468) | 0.615 | 457 (370) | 400 (314) | 0.243 |  |
| **Meeting exercise guidelines^d^**, n (%) | 210 (88) | 55 (87) | 0.115 | 181 (88) | 84 (88) | 0.999 |  |
| **Diagnosis**, *n* (%) |  |  | **0.009** |  |  | 0.654 |  |
| Breast cancer | 194 (82) | 41 (64) |  | 163 (80) | 72 (75) |  |  |
| Prostate cancer | 36 (15) | 20 (31) |  | 36 (18) | 20 (21) |  |  |
| Colorectal cancer | 7 (3) | 3 (5) |  | 6 (3) | 4 (4) |  |  |
| **Primary (neo-)adjuvant treatment**, *n* (%) |  |  | 0.801 |  |  | 0.549 |  |
| Chemotherapy | 125 (53) | 32 (50) |  | 110 (54) | 47 (49) |  |  |
| Radiation therapy | 79 (33) | 21 (33) |  | 68 (33) | 32 (33) |  |  |
| Endocrine therapy | 33 (14) | 11 (17) |  | 27 (13) | 17 (18) |  |  |
| **^a^** p-value for differences between participants included in the analysis and those excluded (independent t-test or Mann-Whitney for continuous variables and Chi2 test for nominal variables),**^b^** Higher scores indicate worse outcome, **^c^**Higher scores indicate better outcome, ^d^ guidelines for cancer survivors, i.e. at least 75 min/week of vigorous intensity aerobic physical activity or 150 min/week of moderate intensity aerobic physical activity or 90 min/week of moderate-to-vigorous intensity aerobic physical activity, and/or two sessions of resistance training/week. Model 1: multiple ordinal logistic regression model estimating baseline predictors of physical activity maintenance; Model 2: multiple ordinal logistic regression model estimating post-exercise intervention predictors of physical activity maintenance. PA: physical activity; SD: standard deviation; HRQoL: health-related quality-of-life; VO_2_max: maximal oxygen uptake; BMI: body mass index; MVPA: moderate-to-vigorous intensity physical activity; IQR: interquartile range. n’s do not all sum to total due to missing data; % is of those with available data. | | | | | | | |
